# Detection of the Strains Induced in Murine Tibias by Ex Vivo Uniaxial Loading with Different Sensors

**DOI:** 10.3390/s19235109

**Published:** 2019-11-22

**Authors:** Emanuele Rizzuto, Barbara Peruzzi, Mariagrazia Giudice, Enrica Urciuoli, Erika Pittella, Emanuele Piuzzi, Antonio Musarò, Zaccaria Del Prete

**Affiliations:** 1 Department of Mechanical and Aerospace Engineering, Sapienza University of Rome, 00184 Rome, Italy; zaccaria.delprete@uniroma1.it; 2 Multifactorial Disease and Complex Phenotype Research Area, Children’s Hospital Bambino Gesù, 00146 Rome, Italy; barbara.peruzzi@opbg.net (B.P.); enrica.urciuoli@opbg.net (E.U.); 3 Sapienza University of Rome, 00185 Rome, Italy; mariagrazia_giudice@hotmail.it; 4 Department of Information, Telecommunication and Electronic Engineering, Sapienza University of Rome, 00184 Rome, Italy; erika.pittella@uniroma1.it (E.P.); emanuele.piuzzi@uniroma1.it (E.P.); 5 Institute Pasteur Cenci-Bolognetti, DAHFMO-Unit of Histology and Medical Embryology, IIM, Sapienza University of Rome, 00161 Rome, Italy; antonio.musaro@uniroma1.it

**Keywords:** strain measurement, digital image correlation (DIC), strain gauges, tissue biomechanics, mechanotransduction, uniaxial loading, bone tissue

## Abstract

In this paper, the characterization of the main techniques and transducers employed to measure local and global strains induced by uniaxial loading of murine tibiae is presented. Micro strain gauges and digital image correlation (DIC) were tested to measure local strains, while a moving coil motor-based length transducer was employed to measure relative global shortening. Local strain is the crucial parameter to be measured when dealing with bone cell mechanotransduction, so we characterized these techniques in the experimental conditions known to activate cell mechanosensing in vivo. The experimental tests were performed using tibia samples excised from twenty-two C57BL/6 mice. To evaluate measurement repeatability we computed the standard deviation of ten repetitive compressions to the mean value. This value was lower than 3% for micro strain gauges, and in the range of 7%–10% for DIC and the length transducer. The coefficient of variation, i.e., the standard deviation to the mean value, was about 35% for strain gauges and the length transducer, and about 40% for DIC. These results provided a comprehensive characterization of three methodologies for local and global bone strain measurement, suggesting a possible field of application on the basis of their advantages and limitations.

## 1. Introduction

Mechanical loading is a powerful physiologic anabolic stimulus for bone [1,2,3,4] and the uniaxial compression of long bones represents a very common technique to activate mechanotransduction [5,6,7]. Mechanotransduction is therefore related to the capability of bone cells to sense mechanical stimuli and convert them into biological responses [8,9,10] At this point, it has to be remarked that an amplification theory based on a multiscale modelling is widely accepted, suggesting that osteocytes (the main mechanical sensors) are not equal in terms of the mechanical stimuli being received. On the basis of their location within the bone tissue they may be subjected to strains even ten time greater than that occurring in vivo. [11,12,13]. 

During the last 10 years, the uniaxial compression of long bones technique was highly employed to evaluate bone adaptation to mechanical compression and to study the role of mechanical load in bone-related pathologies, mainly in vivo [1,6,14]. Recently, the role of bone axial compression has also been approached with ex vivo experiments [3,15]. Whilst this condition deprives the specimen of the whole signaling occurring in vivo, it allows for working in a more controlled system to focus on the tissue of interest. It also enables testing non physiological loadings to obtain a comprehensive characterization of cell mechanotransduction. Finally, the use of the organ culture technique allows keeping the specimen alive for several days, providing a thorough monitoring of the bone’s response to mechanical loading [15]. 

Bone cell mechanotransduction occurs when the loading parameters, namely, amplitude, frequency and resting time, accomplish specific features [7,16,17,18]. Force controlled compressions are most widely employed for these kind of tests, and in particular, the load amplitude must be able to induce local strains higher than a specific threshold (e.g., +600 µe) [2]: an accurate measurement of local strains [19] is therefore fundamental to unraveling the suitable amount of force that has to be applied at the specimen’s ends in order to induce mechanotransduction. The strains measured through the use of micro strain gauges are then employed to calibrate the loading machine: the loading values inducing the suitable strains are identified and used on other specimens of the same animal strain to perform the desired tests. Indeed, due to the specimen complexity, in terms of both shape and composition, it is not possible to devise a general relationship between the applied amplitude and the local strain values. Strain gauges are sensitive and accurate transducers, and their use allows for the measurement of the local strains occurring exactly on the area of interest. On the other hand, their use is invasive and relies on the operator’s capability. To obtain repeatable results, the strain gauge must be glued exactly on the same specimen location and with the very same axial direction each time. Since the surface strain field of tibia specimens is highly inhomogeneous [20], any variation in the strain gauge accommodation yields measurements of different strain values. As a result of this, standard deviation (SD) values of about 30% around the mean value are very common [3,6,20,21]. Nonetheless, this sensor is the most widely used for measuring local strain during in vivo mouse tibia loading. In addition, it has to be remarked that even if cyanoacrylate-based glues are the most suited adhesive for these applications and are widely utilized to attach the strain gauges [3,18,22,23,24], their use can induce a strain loss between the sensor and the specimen [25]. A significant bias in the measurements can be therefore introduced.

Recently, the use of digital image correlation (DIC) was applied to measure, ex vivo, the local strains of tibia specimens subjected to almost quasi-static, uniaxial compression [18,26]. DIC is a contactless technique based on specimen image acquisition for the entire duration of the experiment. The images are then correlated to predict the surface strain field of any desired region of interest (ROI). This technique is gaining more and more interest in the biological fields, given the absence of contact with the specimens and the capability of accurately working with inhomogeneous, anisotropic and nonlinear materials [11,27,28,29]. To date, its accuracy has been evaluated, also in comparison with strain gauge accuracy, for very small region of interest under quasi-static conditions [18] or when testing dynamic loads with large ROIs [30]. 

Within this context, the aim of this work was to compare the two techniques mainly employed to measure local strains when testing bone specimens in the conditions known to induce new bone cell formation [7,15]. As a result of this, we were called to characterize these techniques when working with very small ROIs and high dynamic conditions. If it were demonstrated that DIC has sufficient accuracy to predict bone samples’ longitudinal strains when approaching the study of bone cell deposition in vivo or ex vivo, its use could be taken into account for all those conditions where the strain gauges cannot be employed. Indeed, strain gauges cannot be employed when very small specimens (collected from young mice) must be tested, or when multiple measurements across different days are needed; for example, to test the effects of a specific treatment. A third technique, that takes advantage of a rotary moving coil motor-based length actuator/transducer, was characterized as well to evaluate its usefulness in providing accurate measurements of the specimen global shortening as complementary information.

## 2. Materials and Methods

### 2.1. Animals

All the experiments were conducted within the animal welfare regulations and guidelines of the Italian national law D.L. 04/03/2014, n.26, about the use of animals for research. A total number of 22 C57BL/6 mice (16–20 week old) was employed in this study. The mice were bred in the “conventional clean” animal facility of the DAHFMO-Unit of Histology and Medical Embryology of the University of Rome La Sapienza. The mice were housed in filtered cages within a 70 cage rack with HEPA filter on it. Four mice were housed in each cage and had food and water ad libidum. The bedding utilized was the “Scobis Uno” (Mucedola) made up of ref fir. They underwent a standard 12/12 h light/dark cycle at a room temperature of 22 ± 2 °C. Health status of all the animals was monitored every 4 months, according with the Italian Law. 

The mice were sacrificed by cervical dislocation immediately prior to the experiment. For each animal only one tibia was tested. Two specimens were employed to evaluate strain gauge measurement repeatability, and two for evaluating DIC measurement repeatability. Three specimens were used to study the relationship between the applied load and the local strain measured through strain gauges, and three to study the same relationship when measuring local strains with DIC. Six specimens were employed to evaluate strain gauge accuracy, and six to evaluate DIC accuracy. Of note, since the relative global shortening was measured for all the specimens we tested, the measurement repeatability of the rotator moving coil employed as a length transducer was evaluated on four specimens, the relationship between the applied load and the relative global shortening was studied for six specimens, and the length transducer measurement accuracy was evaluated for twelve tests. 

### 2.2. Experimental Set-Up

The experimental system employed to compare the 3 measurement techniques was based on a dual mode force/length actuator/transducer (305C-LR–Aurora Scientific Inc., Aurora, ON, Canada) driven by a personal computer equipped with a data acquisition board (PCIe-6353–National Instruments, Austin, TX, USA). Images were acquired by a fast speed camera (acA2040-180km–Basler, Ahrensburg, Germany) mounted on a stereomicroscope (SMZ 800–Nikon, Tokyo, Japan) with a 0.5 X Plan Apo lens and connected to an image acquisition board (PCIe-1433–National Instruments, Austin, TX, USA). Strain data signal was conditioned with an amplifier system (2100–Vishay, Malvern, PA, USA) equipped with a bridge completion module (2120–Vishay, Malvern, PA, USA). 

**Figure 1:**
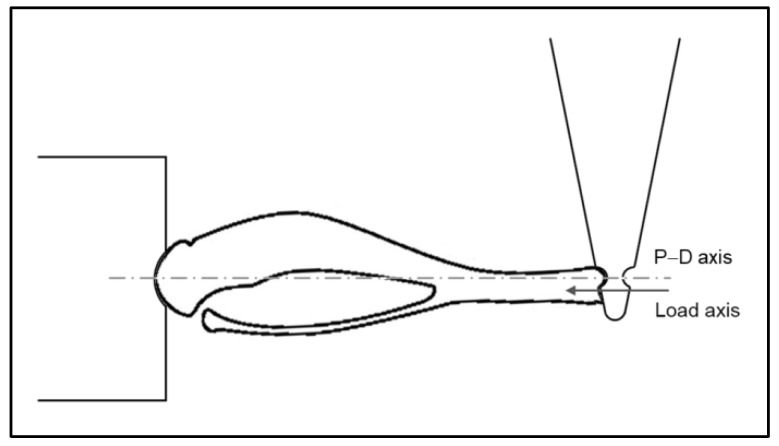
Scheme of the fixing method employed to uniaxially load bone specimens. P–D axis is the proximal-distal axis.

The specimen to be tested was mounted between the lever arm of the 305C-LR motor and a fixed clamp properly shaped to accommodate the proximal head of the tibia, as shown in Figure 1. The tip of the lever is also shaped to accommodate the distal end of the tibia. A 3-axis micro-manipulator was used to precisely align the specimen, so that the force vector was superimposed to the proximal–distal (P–D) axis [21] of the tibia. Of note, this choice caused the medial surface of the tibial midshaft to be perpendicular to the lens, so that the region of interest focused by the camera resulted in plane. The ASI 305C-LR allowed us to uniaxially load the bone specimen with force controlled signals up to 10 N with a resolution of 1 mN, and to simultaneously measure length and force with resolutions of 1 µm and 1 mN respectively. The entire system was placed on a vibration isolation bench (Newport Vision IsoStation).

A software developed in LabVIEW 2012 was used to set all the parameters of the loading signal, i.e., the shape, frequency, and amplitude, and to choose the acquisition frequency for the strain data and the images. For all the tests reported in this work, the specimens have been subjected to triangular loads of different amplitudes and frequencies, and to a compression signal which summarizes the features of the mouse step, herein labelled as “step signal” [7,31]. This signal is composed of a load ramp of 0.075 s duration followed by a symmetric unload ramp, with a rest period of 0.100 s between each load cycle; loading was applied at 4 cycles/second, and this is the signal generally employed to induce mechanotransduction in tibia specimens in vivo.

### 2.3. Compression Tests

All the tests performed in this work were based on force controlled compression loads [7,14]. Since the strains occurring on the tibia medial surface cannot be directly related to the applied compression (whether controlled in force or in position), the use of force controlled signals is preferable since they better reproduce the in vivo conditions. To ensure that the specimen was properly fixed on the experimental system, a preload of 0.5 N was applied before starting each experiment and the resulting tensional status was taken as the reference.

To evaluate the measurement repeatability of the sensors and techniques of interest, we proceeded as follows: we subjected 4 different specimens to 10 triangular compression signals of 9 N of amplitude and to 10 step signals of 9 N of amplitude, with a resting of 90 s before each of them. Two specimens were used to assess the strain gauge repeatability, and 2 for the DIC repeatability. The repeatability of the length transducer in measuring the relative global shortening was evaluated on the 4 samples. 

To evaluate the relationship between the applied load (F) and the measured longitudinal local strain (ɛ_x_), 6 specimens were subjected to triangular compression signals of 1 N, 3 N, 6 N, and 9 N of amplitude at 0.1 Hz, 0.5 Hz, 1 Hz, and 4 Hz, and to step signals of 1 N, 3 N, 6 N, and 9 N of amplitude, randomly delivered with 90 s of resting time before each of them. Three specimens were equipped with a micro strain gauge for the measurement of the longitudinal local strain and three were strewed with a silicon-carbide powder (see Section 2.4 for details) for DIC measurements. The relationship between F and the relative global shortening (Δl/l) was studied for all the 6 tested specimens. 

Finally, we tested 6 + 6 specimens to evaluate the measurement accuracy for strain gauge and DIC techniques, by focusing on the highest value of force already selected, 9 N. The length transducer measurement accuracy was indeed evaluated on all the 12 tested specimens. Of note, 9 N is a value shown to be considerably higher than that required to induce bone cell activation (usually 3 N) [7,14]. Once having demonstrated the linear relationship between F and ɛ_x_, and between F and Δl/l, the highest force value sums up all the others. Moreover, the correlation algorithm employed in this work [32] was specifically designed to extend the correlation procedure to a set of consecutive pictures, across which the path of a point was considered to be smooth according to a chosen spatiotemporal function. In view of this, the algorithm predictions for 1 N, 3 N, and 6 N would be more accurate when loading the specimens up to 9 N. The bone samples were therefore subjected to the following protocol: one step signal of 9 N of amplitude followed by 4 triangular compressions of 9 N at the following frequencies, 0.1 Hz, 0.5 Hz, 1 Hz, and 4 Hz, which correspond to loading speeds of 120 N/s (for the step signal), 1.8 N/s, 9 N/s, 18 N/s, and 72 N/s respectively. It is worth noting that, except for the step signal that was always delivered at first, the other signals were applied randomly to avoid adaptation. An extra step signal was then delivered at the end of the protocol for a double check of tissue preservation. The strains measured by applying the last and the first step signals were indeed compared. For each test, the actual value of force applied to the specimen and provided by the actuator/transducer was considered for computations, instead of the theoretic value. 

According to the above-described protocols, each specimen was tested for no longer than 40 min. At this point, it has to be remarked that when characterizing a technique employed for measurements of biological specimens, a crucial point is related to the variability introduced by the preservation of the tissue, its humidity, and its hydration conditions. 

### 2.4. Local Strain Measurement

When a compression load is applied on a tibia specimen, the midshaft encounters a tensile strain due to the specimen’s arched shape. We tested two different methods of measuring this local tensile strain, the use of a strain gauge and the digital image correlation technique.

In the first case, the longitudinal strain was measured through a commercial micro strain gauge with a sensitive area of about 0.2 mm^2^ (EA-06-015LA-120; Micro-Measurements, Wendell, NC, USA). The strain gauge was glued on the medial surface of the tibial midshaft aligned with the bone’s long axis using cyanoacrylate glue [22], close to the fibula attachment, where there was still sufficient flat surface to accommodate it. Attention was taken in placing the gauge as parallel as possible to the tibia medial side. During all the tests, the samples were kept out of the physiological bath to be consistent with in vivo experiments. Indeed, during the dissection procedure and the resting times, the specimens were always kept in a Dulbecco’s phosphate buffer physiological solution. The strain gauge output was connected to a signal conditioning amplifier (2120 module–Vishay, Malvern, Pennsylvania, USA) and the strain values were recorded simultaneously with force and length signals. For each test, the strain gauge was carefully calibrated to gather the strain scale factor by using the internal shunt resistors provided by the 2120 module.

**Figure 2:**
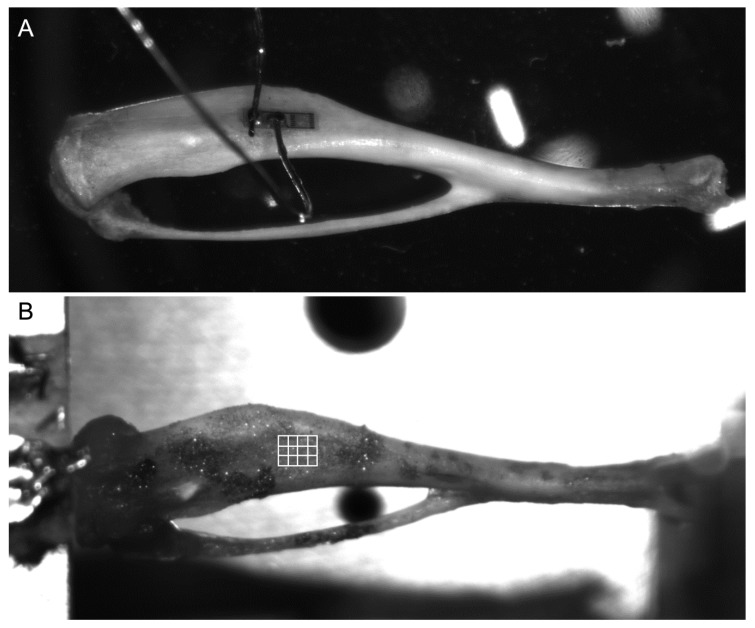
The micro strain gauge was placed on the medial surface of the tibial midshaft (**A**), and the digital image correlation (DIC) region of interest was selected to be as superimposable as possible to the gauge active grid (**B**).

To test the digital image correlation as a technique to measure tibia local strains, another set of specimens were subjected to the same compression tests, and the images of the tibial midshaft were continuously acquired to be correlated through the use of an original correlation algorithm, previously optimized for working with biological tissues [33]. To enhance the algorithm correlation capability, the samples were strewed with a silicon-carbide powder to obtain a suitable speckle pattern made up of 40 μm diameter particles (357391–Sigma-Aldrich, St. Louis, MO, USA) [33]. The images were acquired with a fast speed camera at the resolution of 2040 × 1020 pixels, and the speed of acquisition was set up proportionally to the frequency of compression to keep constant the spatial distance between two consecutive images for each test. In particular, the camera speed of acquisition was determined by the following equation: f_fps = 30 x f_Hz, where f_fps is the camera frequency of acquisition and f_Hz is the signal frequency. The pixel dimension is of 5.5 × 5.5 µm. It must be noted that the exposure time was kept fixed at 8270 µs (the greatest value that could be used at the highest acquisition speed) to ensure an equal amount of light at the sensor for all the tests, making the correlation algorithm work in the same experimental conditions. As for the previous set of experiments, the samples were kept out of the physiological bath. A region of interest of 5 × 4 nodes with a subset size of 25 pixels (corresponding to an area of about 0.23 mm^2^) was selected to be almost superimposable to the place where the strain gauge was expected to be glued, as shown in Figure 2, and the 2-dimensional surface strain field was predicted. 

### 2.5. Relative Global Shortening Measurement

Along with the local strain measurement, the bone global shortening was measured as well. The displacement of the lever arm tip was continuously acquired at 1000 Hz for the entire duration of the test, and the relative global shortening (Δl/l) was obtained by dividing the length change, a shortening, by the tibia initial length L_0_. To take into account the compliance of the testing machine, thus trying to reduce at the minimum the “end-artifact” [34], we measured in advance the lever arm tip displacement when loading a stainless steel cylindrical specimen with the same 4 force values employed to test bone specimens. The length and diameter of the stainless steel specimen were close to that of the bone tested, and the tip displacement measured in this way was subtracted from the values measured during the experimental tests. 

L_0_ was measured by optical comparison with a calibrated grid (Edmund optics). The accuracy of the above-described grid was ±0.001 mm for 2 adjacent lines. The accuracy in the measurement of the distance between 2 lines of the grid was hypothesized to be of ±2 pixels (±11 µm) for each side, while the accuracy in the measurement of tibia length was hypothesized to be of ±4 pixels (±22 µm) for each side, since the exact end of the specimen was less defined than that of the calibrated grid. As a result of this, the total accuracy of the optical procedure was of ±34.8 µm. Since the specimens employed in this work were longer than 17 mm, the accuracy error due to length measurement with the optical method was always lower than 0.2%. The relative global shortening was measured for all the tests, and the results were reported as absolute values. 

### 2.6. Statistics

The data obtained by loading the specimens with different load amplitudes were linearly fitted with Matlab R2014b. The same tool was used to fit the local strain response to a single compression signal. The linear fitting was always enforced to pass through the origin since for a null load a null strain should theoretically be measured. Differences in the values of the induced strains for different loading frequencies were evaluated with a 1-way ANOVA by using GraphPad Prism 6.0, and differences were considered significant when *p* < 0.05. 

## 3. Results

For all the proposed techniques, measurement repeatability was computed by subjecting the specimens to repetitive triangular loading signals and then to repetitive step signals. The coefficient of variation (CV), i.e. the ratio between SD and the mean value, was computed across the ten repetitions for both loading signals (triangular and step) for each transducer/technique, and resulted almost identical in the two compression signals. When using the strain gauge to measure longitudinal local strain, the CV obtained through the ten repetitive compressions was always lower than 3%, while it resulted in the range between 7% and 10% when using the DIC. A CV in the range between 7% and 10% was also obtained when measuring relative global shortening through the use of the length transducer. 

**Figure 3:**
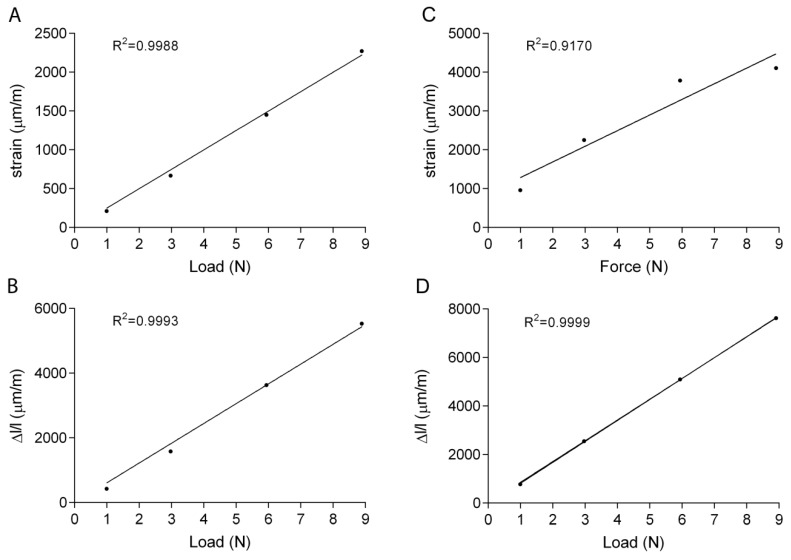
Experimental values and linear interpolation of a representative test performed loading the specimen with a step signal, and measuring the strain with the micro strain gauge (**A**) and DIC (**C**). The global shortening was measured with the length transducer for the strain gauge (**B**) and DIC (**D**) tests.

Figure 3A shows an example of the relationship between the applied load and the longitudinal local strains measured with the micro strain gauge. As expected, this relationship is highly linear, with R^2^ values always greater than 0.98. Figure 3B shows the relationship between the applied load and the longitudinal global shortening obtained for the same test: despite the very particular shape of the specimen this relationship was found to be highly linear, with R^2^ values always greater than 0.99. On the other hand, the DIC technique was able to provide accurate measurement of the longitudinal local strain only when the specimens were loaded with the highest loads (i.e., higher strain values) [29,35]. Since the ROI was determined to be almost coincident with strain gauge’s active area, we chose the subset size to increase the accuracy for loading levels between 3 N and 6 N, the ones mainly employed to induce bone cell activation in vivo [6,7,14]. When the specimens were loaded with 3 N, the DIC provided accurate results for about one half of the tests, while it was mostly unsuccessful for the measurement of local strains of specimens loaded at 1 N. However, when the correlation was successful for all the tested loads, or at least for three of them, the relationship between the applied load and the longitudinal strain was found to be highly linear, with R^2^ values always greater than 0.9. Figure 3C shows an example of the relationship between the load applied and local strain measured with DIC for one of the cases in which the correlation was successful for all the four input loads. Figure 3D shows the corresponding global shortening.

**Figure 4:**
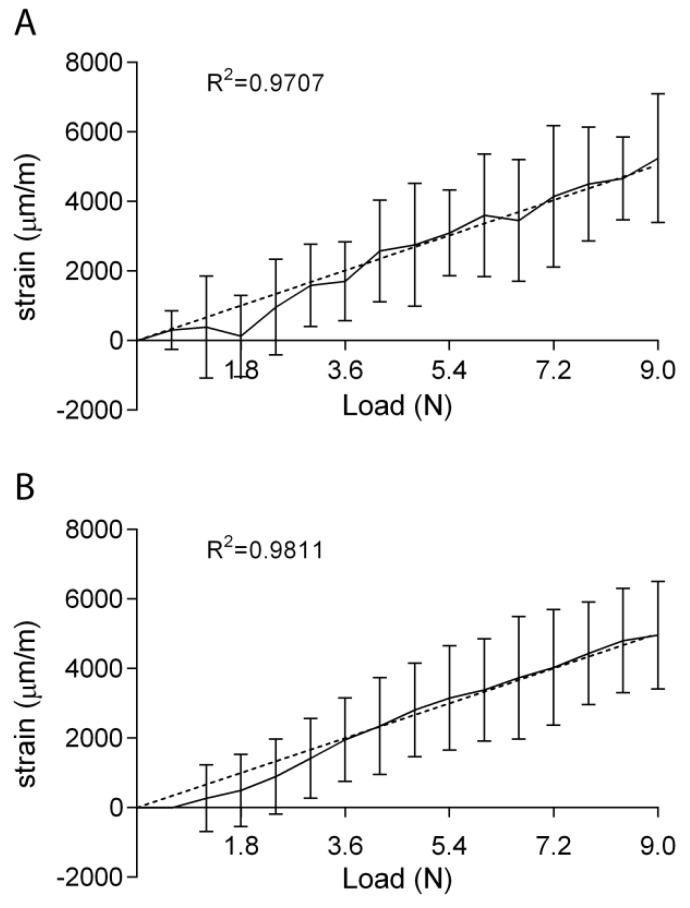
Means ± SDs of the values measured by DIC when loading the specimens with a 4 Hz triangular load of 9 N (**A**). A one-point moving average filter allowed the relationship to be more linear (**B**) *n* = 3.

Once having characterized the relationship between the applied load and the measured strain, we proceeded with evaluating the accuracy of the three techniques by focusing on the highest value of force, 9 N. By applying this value it is possible to gather information for all the smaller loads. However, a deeper analysis of the longitudinal strain measured with the DIC with loads of 9 N was necessary. Figure 4A shows the average longitudinal strain measured with DIC when the specimens were loaded with a 4 Hz triangular signal. This result confirmed that the relationship between the applied load and the longitudinal strain is measured to be linear for increasing loads, with lower accuracies for the lowest load values. The choice of measuring the accuracy for the three techniques by applying the highest load was therefore confirmed to be correct. It has to be kept in mind that DIC is not able to provide accurate results when measuring smaller strain values. The use of a one-point moving average filter allowed obtaining a good linear relationship from 3.6 N (i.e., 0.05 s) on Figure 4B.

**Figure 5:**
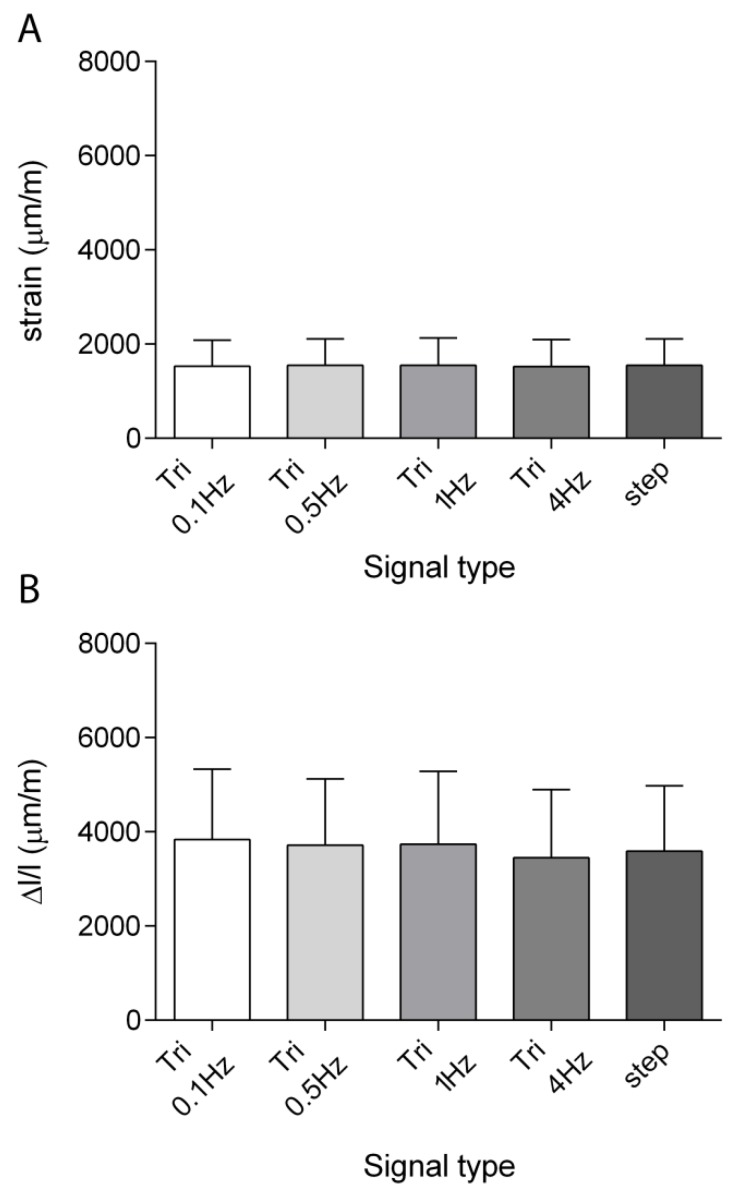
Means ± SDs of the longitudinal strains measured with the micro strain gauge (**A**) and of the global shortening measured through the length transducer (**B**) *n* = 5.

Figure 5A displays the longitudinal local strains measured with the micro strain gauges when subjecting the specimens to the 9 N compression force, and Figure 5B shows the relative global shortening measured during the same tests. The magnitudes and standard deviations measured with the two techniques showed similar results for all the tested frequencies. The coefficient of variation was of about 35%, on average, for the measurements performed with both the strain gauges and the relative global shortening.

**Figure 6:**
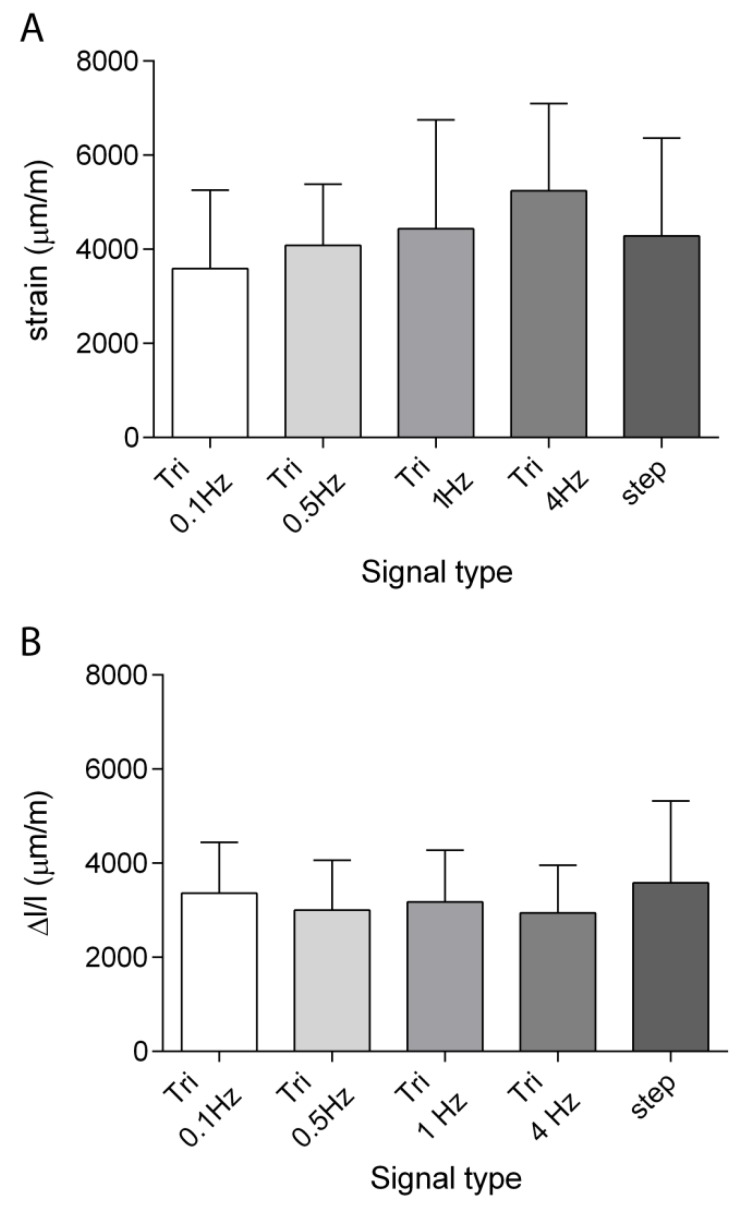
Means ± SDs of the longitudinal strains measured by DIC (**A**) and of the global shortening measured with the length transducer (**B**) *n* = 5.

Figure 6A displays the longitudinal local strains measured by DIC when subjecting the specimens to the 9 N compression force, and Figure 6B shows the relative global shortening measured during the same tests. Again, the relative global shortening magnitudes and standard deviations showed similar results for all the tested frequencies, and were in good agreement with the values obtained during the previous set of experiments (Figure 5B). On the other hand, the local strains measured with DIC had a more variable pattern for the tested frequencies, even if the differences were not statistically significant (*p* = 0.68). The standard deviation values were basically constant for all the frequencies we tested, and their ratio to the mean values was of about 40% on average. It is interesting to note that the longitudinal local strain values measured through DIC were about the double of those measured with the strain gauges. 

**Figure 7:**
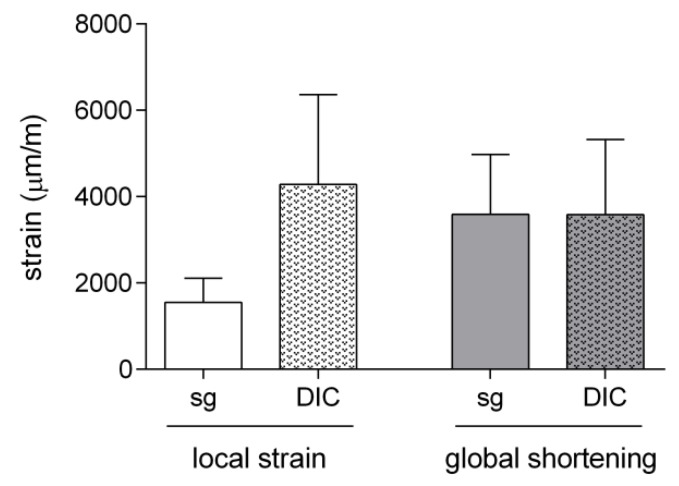
Means ± SDs of the local and global strains measured by strain gauges (sg) and DIC and by the length transducer during the step signal input *n* = 5.

Figure 7 shows, as an example, the comparison of local and global strains measured during the 9 N step signal input with the three different techniques. Longitudinal local strains measured through the strain gauges were measured to be about half of the longitudinal local strains measured with DIC, while the relative global shortening was measured to have about the same mean value and SD for experiments involving strain gauges and DIC.

**Figure 8:**
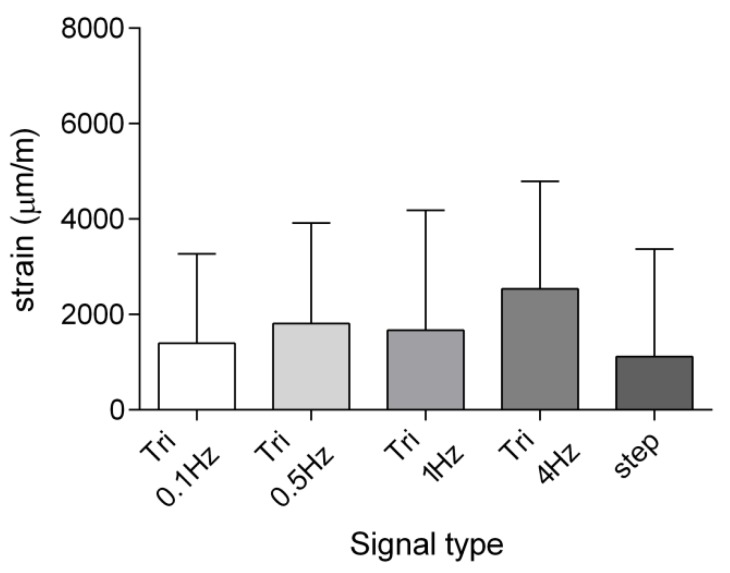
Means ± SDs of the transverse strains measured by DIC *n* = 5.

The use of digital image correlation also allowed predicting the transverse local strains, as shown in Figure 8 for the 9 N compression force. Interestingly, these results highlighted that tibia underwent a positive deformation (enlargement) along the transverse direction as well, when compressed, with a trend that is in accordance with that measured for the longitudinal strain. Once again, this outcome is probably related to the particular shape of the tested specimen. However, it has to be noted that CV was of about 135%, on average.

## 4. Discussion

The measurement of local strains is fundamental when dealing with bone cell mechanotransduction in vivo and ex vivo, and the knowledge of the measurement accuracy is therefore crucial. The use of micro strain gauge, actually the gold standard sensor, was compared with the digital image correlation predictions to capture the longitudinal local strains of the tibial midshaft surface. In parallel, the measurement of tibia global shortening obtained with a rotary moving coil motor based length transducer was characterized as well.

A critical point that has to be addressed when dealing with biological tissue is the measurement repeatability. Biological specimens are live tissues, and their properties, including mechanical ones, continuously change. When approaching ex vivo experiments, other issues related to tissue preservation or the humidity and dehydration conditions may arise to affect the measurements [36]. Indeed, in this work the specimens were subjected to experimental protocols shorter than 40 min, but the tissues were not immediately tested. When measuring the strain gauges to measure the local strain, a certain amount of time (about 30 min) was necessary for the right placement of the strain gauges and the wire soldering, while when using the DIC we waited the same amount of time for consistency. 

The measurement repeatability we measured with DIC was just a little bit higher than that proposed in the literature for ex vivo experiments, with CV values that ranged from 7% to 10% compared to average values of 6% [26]. This discrepancy may be ascribed to the different speeds of compression, and in turn, to the different image acquisition frame rates. Carriero et al. [26] loaded the specimens with quasi-static signals (0.133 N/s), while we used dynamic signals (from 1.8 N/s to 120 N/s) to test these methodologies in the exact conditions that can induce bone cell mechanotransduction. The CV of the measurements performed with strain gauges was found to be of 3% on average. By testing a synthetic model of femur, Cristofolini et al. [37] obtained a strain gauge CV lower than 1%. In our opinion, the small difference we found was due to the biological nature of the tissue we tested, and the big difference in the strain gauge dimensions employed, that may have affected the sensor optimal placement. Indeed, our results confirmed a very high repeatability of this transducer in these particular experimental conditions, and pointed out that our experiments were performed in a steady state of tissue dehydration [38]. A result close to that of DIC was obtained for the measurement repeatability of the global shortening. Again, the fact that the global shortening measurement repeatability was found to be equal in the two sets of experiments (those with the use of strain gauges and those involving DIC) confirmed that all our tests were performed in a steady state.

The relationship between the applied load and the local longitudinal strain, and the relationship between the applied load and the global shortening were found to be highly linear in the range of force usually employed to induce in vivo mechanotransduction in tibia samples. This outcome was already known for the relationship between the applied load and the local strain [3,7,14,20], but nothing could be expected for the relationship between the applied load and the global shortening. The particular shape of the specimen could have led to non-elastic response or to hysteresis phenomena. This outcome supports the feasibility of the global shortening to be considered as a parameter to complement the standard local strain measurement when dealing with cell activation of bone specimens. Of note, as already known, the DIC technique was not able to provide accurate results when the specimens were compressed with low values of load; i.e., for small values of local strain. At that point, the correlation algorithm we employed was designed to provide more accurate values when the correlation procedure was extended to a set of consecutive pictures, across which the path of a point was considered to be smooth according to a chosen spatiotemporal function. The use of high loading compression signals (i.e., 9 N) indeed allowed obtaining more accurate values with DIC. Even if this is a limitation of this technique, it has to be noted that the strain values able to activate the quiescent bone cells are always higher than 300 µɛ and up to 2000 µɛ [2,7,14] when measured through the strain gauge. These values are obtained with compression signals of at least 2–3 N.

The longitudinal strain values measured with the micro strain gauges are in accordance with literature data for in vivo [2,14,20,24] and ex vivo [3] tests. Regarding the use of DIC to predict longitudinal strains, Sztefek et al. [18] reported a good agreement between the outcomes obtained with DIC and strain gauges. Gustafson et al. [30] reported a normalized error of about 10% when measuring vertebral body deformations using strain gauges and DIC. The normalized error obtained with our measurements is of about 45% on average. In our opinion, there are several causes that may have led to this high difference in the values we measured with strain gauges and DIC. A local strain loss caused by the cyanoacrylate glue under the strain gauge could have occurred, as suggested by Komurlu et al. [25]. Depending on the cyanoacrylate thickness, they measured differences in the strains of the sample and the gauges up to about 25%, and it is plausible that porous materials such as bone may even enhance this issue. The use of highly dynamic signals compared to the quasi-static signals employed by Sztefek et al. could, therefore, partially explain the difference in the two outcomes. Indeed, they also provided lower values of longitudinal strains measured with the strain gauge. It is worth noting that we used very small strain gauges, and therefore small ROIs, that could have caused higher differences in the results obtained with the two methodologies, than when using bigger strain gauges and ROIs [30,37]. Indeed, tibia surface strain field is known to be highly inhomogeneous [20] so that even a small difference between the selected ROI and the exact zone were the strain gauge was supposed to be glued may introduce high errors, whereas the use of bigger strain gauges (and larger ROIs) may help to decrease this issue since the outcome is the results of an average process on a bigger surface. The effect of reinforce of the strain gauge itself is another issue that might have induced alterations in the measured strain, giving the small dimensions of the sensor and the use of dynamic loads. It has also to be noted that, during our ex vivo experiments, we loaded the specimens along their P–D axis [21] to increase the repeatability of the measurements, since in our experimental configuration we could not take advantage of the knee and the foot as supports. The differences in the strains can be therefore ascribed only to the specimens’ shapes, but these values can differ from those measured in vivo, or in those experiments where the ankle and the foot were maintained as well. 

The measurement variance obtained with the use of micro strain gauges was in accordance with the literature [3,6,20], highlighting that this high variability is related to the strain gauge itself and to its placement and not to the experimental conditions. The tibia surface strain field is highly inhomogeneous [20,21], and even small differences in the sensor placement or in the choice of the ROI may lead to very different strain values. Of note, when the strain gauge location was verified on subsequent microCT images, the CV was just a little bit lower [7]. The measurement variance of DIC predictions was found to be slightly higher than that of the strain gauges. Again, this variability is related to the fact that even if the ROI is chosen to be as similar as possible for all the specimens, it undoubtedly refers to different zones of the tibia, and to different orientations. On the other hand, DIC is a contactless technique which allows testing the specimens in their unaltered state. For example, it could be employed in those conditions in which the strain gauges cannot be used; e.g., when testing very small specimens, or when measurements across different days are necessary, both in in vivo and ex vivo conditions. Finally, DIC allows for measuring the entire 2-dimensional strain field, potentially for the entire specimen. 

The measurement variance obtained through the length transducer was found to have a measurement variance similar to that of strain gauges, being that error mostly related to the exact positioning of the loading axis. In addition, the global shortening was found to return reliable values for low loading forces as well, in contrast to DIC, providing a more linear behavior. Considering that global shortening can be easily computed when a length actuator/transducer is employed to induce tibia loading, its use can be taken into account to provide useful information on the measurement of local strains. 

Finally, it has to be noted that the lowest value of CV we obtained for the measurement accuracy of all the tested methodologies was of about 35% on average, confirming how big is the variability that has to be expected even among “identical” biological specimens. For local and global strains no statistically significant differences were reported across the tested frequencies.

## 5. Conclusions

In this paper we characterized two different methodologies to measure, ex vivo, the local strains induced by the uniaxial compression of mouse tibia specimens in the experimental conditions that yield to bone cell mechanotransduction. The first technique is based on the use of micro strain gauges that, to date, represents the gold standard sensor; the other is a technique that has gained more and more attention in the biological field in the last years, the digital image correlation (DIC). In addition, we exploited the use of a rotary moving coil motor-based length transducer to measure tibia global shortening as a technique to provide complementary information of bone remodeling. Our results confirmed that the measurement of longitudinal local strains through the use of micro strain gauges is affected by a quite high variance. The measurement of the same local strain by DIC is affected by a variance just a little bit more; on the other hand, DIC may be used when strain gauges cannot be applied; for example, when testing small specimens or when multiple measurements are necessary in both ex vivo and in vivo long-term treatments. The normalized error between the strains measured with the strain gauges and the DIC was about 45%, pointing out that several issues may affect these kind of measurements, as a strain loss along the cyanoacrylate interlayer, the positioning and alignment of such small strain gauges and ROI, the strain gauge reinforcement, and moisture. Finally, the relative global shortening was shown to return a variance of the measurements similar to that of the strain gauges and to have a high linear relationship with the applied load. A comprehensive characterization of the main techniques and sensors used to capture the mechanical parameters during bone cell remodeling in bone axial loading is of fundamental importance, and may cause other researchers to choose the most suitable methodology for each experimental condition, with a proper knowledge of its advantages and limitations.
